# Preparation and effect of lyophilized platelet-rich fibrin on the osteogenic potential of bone marrow mesenchymal stem cells in vitro and in vivo

**DOI:** 10.1016/j.heliyon.2019.e02739

**Published:** 2019-11-01

**Authors:** Zhifa Wang, Leng Han, Tianyu Sun, Weijian Wang, Xiao Li, Buling Wu

**Affiliations:** a School of Stomatology, Southern Medical University, Nanfang Hospital, Southern Medical University, Guangzhou, 510515, PR China; b Department of Stomatology, General Hospital of Southern Theater of PLA, Guangzhou, 510010, China; c Department of Pathology, General Hospital of Southern Theater of PLA, Guangzhou, 510010, China

**Keywords:** Bioengineering, Cell biology, Materials science, Molecular biology, Stem cell research, Lyophilized platelet-rich fibrin, Bone marrow mesenchymal stem cells, Cell sheet, Bone tissue engineering

## Abstract

**Objectives:**

The goal of this study was to prepare lyophilized platelet-rich fibrin (L-PRF) and analyze the combined use of L-PRF and osteogenic bone marrow mesenchymal stem cell (BMSC) sheet fragments for bone tissue engineering via in vivo injection.

**Methods:**

First, fresh PRF (F-PRF) was lyophilized to prepare L-PRF, the characteristics of which were examined through gross morphological, and histological and microstructural observations. In addition, the kinetics of growth factor release from L-PRF and F-PRF were also determined by enzyme-linked immunosorbent assay (ELISA). Subsequently, after assessing the proliferation and osteogenic differentiation of BMSCs exposed to L-PRF or F-PRF in vitro, we subcutaneously injected BMSC sheet fragments with L-PRF or F-PRF into nude mice and assessed bone formation through microcomputed tomography and histological analyses.

**Results:**

We observed that L-PRF released growth factors that favored BMSC proliferation and osteogenic differentiation in vitro. The combined use of L-PRF and osteogenic BMSC sheet fragments enabled bone tissue regeneration in vivo, and no significant difference between the F-PRF and L-PRF groups was observed (P = 0.24).

**Conclusions:**

The results of this study demonstrate that the combined use of L-PRF and osteogenic BMSC sheets may have potential in the fabrication of engineered bone.

## 1 Introduction

The treatment of nonunion and bone defects remains a common clinical problem [1]. In recent years, rapid developments in tissue engineering and regenerative medicine have provided promising strategies for the repair of bone defects [2]. However, transplanting tissue-engineered bone still requires an open and traumatic surgery [3]. Compared with traditional surgical treatments, such as autologous bone grafts and distraction osteogenesis, an injection approach for bone repair involves a simple, minimally invasive, and low risk procedure that significantly reduces scar formation and allows for repeatability [4, 5, 6].

Tissue-engineered bone therapies are commonly divided into two major categories, scaffold-based and scaffold-free cell therapies [7]. A variety of scaffold –based materials have been employed for bone regeneration to deliver cells and other signals providing 3D environment to facilitate cell communication or synthesize bone specific matrix [7]. Scaffold commonly used in tissue engineering include biodegradable polymers of either synthetic or natural origin, such as collagen, alginate, hydroxyapatite (HA), and tricalcium phosphate (TCP) and so on. However, scaffold materials have some shortcomings, including their potential immunogenicity, inflammatory response, toxicity of degradation products, unpredictable gelation kinetics, and unsatisfactory regulation of the degradation rate [8, 9]. Scaffold-free cell therapies, such as cell sheet technologies without additional need of scaffold, were used as alternatives to scaffold-based cell therapies prior to the development of the ideal scaffold material [10, 11, 12]. Ma et al. observed that bone marrow mesenchymal stem cell (BMSC) membranes contain a large number of active cells, an endogenous extracellular matrix (ECM) secreted by the active cells, and relatively complete intercellular connections. A bone-like structure forms after BMSC membranes are injected into the subcutaneous space of nude mice [13]. The speed and effectiveness of noncontinuous bone healing of the mandible can be significantly improved by injecting MSC membrane fragments [13, 14]. In addition, Gruene et al. used computer-assisted MSC deposition technology to print high-density MSC suspensions in vitro and form a bone-free structure without scaffolds [15]. Thus, the development of a scaffold-free cell transplantation technique based on cell sheets indicates that BMSC sheets can be used to generate injectable tissue-engineered bone [16, 17]. Furthermore, compared to dispersed cells, cell aggregates have been shown to significantly improve cell utilization and ease of operation [18].

Platelet-rich fibrin (PRF), a second-generation platelet-rich aggregate, is simple to prepare and gradually releases cytokines during its slow degradation [19]. Therefore, PRF has an excellent ability to promote wound healing and tissue regeneration [20, 21]. The results of our previous study confirmed that PRF significantly promotes the osteogenic ability of BMSC membranes in nude mice and demonstrated the synergistic effect of the combination of PRF and osteoblastic BMSC membranes on bone defect repair in a rabbit skull defect model [22]. The effect of PRF on tissue repair is primarily attributed to cytokine stimulation and fibrin scaffolding. The cytokines in PRF primarily include transforming growth factor beta 1 (TGFβ-1), insulin-like growth factors (IGFs), vascular endothelial growth factor (VEGF) and platelet-derived growth factors (PDGFs), which effectively recruit cells that are important for tissue repair to the defect sites and then promote and regulate the tissue repair process [23, 24]. Furthermore, the fibrin in PRF also provides a three-dimensional structure for stimulating the proliferation and differentiation of recruited cells [25]. PRF has been widely used in bone and cartilage defect repair, oral implants, and severe periodontitis, achieving satisfactory therapeutic results [26, 27]. However, fresh PRF (F-PRF) is commonly limited to use immediately after preparation and autologous applications, making it difficult to store and commercialize for long periods of time. To address the storage issues and extend the clinical applications of F-PRF, a number of studies have investigated the use of freeze-dried PRF and its applications [28]. Freeze-drying is a common method that improves the stability of proteins for tissue regeneration and facilitates the long-term preservation of these proteins [29, 30]. Lyophilized proteins not only have good stability and storage potential but also retain their original biological activity. Although several studies have investigated the use of freeze-dried PRF in recent years, but there is no unified conclusion between the biological characteristics of F-PRF and lyophilized PRF (L-PRF). Therefore, the aim of this study was to investigate the preparation of L-PRF and analyze the combined use of L-PRF and osteogenic BMSC sheet fragments for bone tissue engineering via in vivo injection.

## 2 Materials and methods

### 2.1 Ethical approval

This study was reviewed and approved on July 15, 2018 by the Institutional Animal Care and Use Committee of the School of Stomatology at Southern Medical University (SMU), Guangzhou, China [project identification code: 2015 (kq-034)].

### 2.2 Preparation of lyophilized PRF

PRF was prepared according to a previously reported method with minor modifications [22]. Briefly, 10 mL of blood (in dry, 10-mL Monovette tubes without anticoagulant or other additional chemical agents) was obtained from rabbits and centrifuged immediately for 10 min at 3,000 rpm in a laboratory centrifuge (TDZ5-WS, XIANGYI, Hunan, China). The centrifuged product consisted of three layers, with the PRF clot being located in the middle layer. The PRF clot was harvested with forceps and gently pressed into a membrane between two sterile pieces of gauze using soft compression for 10 s to maintain the wetness of the membrane. The fresh PRF membrane was considered F-PRF, half of which was frozen overnight in a cryorefrigerator at -80 °C. Before lyophilization, the cold trap temperature was reduced to -50 °C and then preheated for 10 min. The samples were quickly transferred from the cryorefrigerator to a vacuum freeze dryer (Alpha1-4 LSCplus, Christ Company, Germany) that was used with the following parameters: a cold trap temperature of -45 °C ± 5 °C, an initial vacuum pressure of 0.1 mbar, a primary drying time of 17 h, a final vacuum pressure of 0.001 mbar, and a secondary drying time of 8 h. After lyophilization, L-PRF was collected, sealed and stored at room temperature for 2 weeks. Finally, the obtained F-PRF and L-PRF membranes were cut into fragments (a few millimeters in diameter) in sterile dishes for further use. Both the F-PRF and L-PRF membranes were prepared for histological examination and observation by scanning electron microscopy (SEM) (S-4800, Hitachi, Japan) and transmission electron microscopy (TEM) (JEM-1230, JEOL, Japan). To reduce the individual differences in PRF from different donors, PRF from eight rabbits was mixed together for subsequent experiments.

### 2.3 Cell isolation and culture

Rabbit BMSCs were isolated and cultured as reported previously [22]. New Zealand rabbits (male, 4 months old with an average weight of 2.5 kg) were obtained from the animal holding unit of SMU. Bone marrow samples were harvested from the rabbits according to procedures approved by the Institutional Animal Care and Use Committee of SMU. Briefly, BMSCs were isolated from the iliac marrow of the adult rabbits. Iliac bone grafts were divided in half, and the marrow was flushed out with low-glucose Dulbecco's modified Eagle's medium (DMEM) (Gibco, Carlsbad, CA) supplemented with 10% fetal bovine serum (Gibco), 0.272 g/L L-glutamine (Sigma-Aldrich, St. Louis, MO), and 2% antibiotics (200 mg/mL penicillin and 200 mg/mL streptomycin; Gibco). Clumps of cells were dispersed to achieve a homogeneous cell suspension by repeated pipetting. Then, the cell suspension was plated in 100-mm cell culture dishes, and standard medium was added to reach a volume of 15 mL. The cells were incubated at 37 °C in 5% CO_2_ and 100% humidity. After 3 days, the medium and all floating cells were removed, and new medium was added to the remaining adhered cells, which were considered to be BMSCs. The medium was changed every 3 days until the cells reached confluence, after which the cells were subcultured at a ratio of 1:3. To reduce the individual differences in BMSCs derived from different donors, BMSCs from 5 rabbits were mixed together for, with cells from the second passage used for further experiments.

Cell sheets were prepared by seeding second-passage BMSCs into 100-mm dishes at a density of 5×10^4^ cells/cm^2^ and adding osteogenic medium that consisted of standard medium supplemented with 50 mg/mL L-ascorbic acid 2-phosphate, 10 mM B-glycerophosphate, and 100 nM dexamethasone (Sigma-Aldrich). The cells were continuously cultured for 2 weeks without passaging, and the osteogenic medium was replaced every 2 days during this period. After 2 weeks of culturing, a thin sheet formed that was easily detached with a cell scraper. Small pieces of the cell sheets were processed for histological examination and observation by SEM. The obtained sheets were cut into small fragments (up to 1 mm in diameter) using a scalpel and suspended in 1 mL of serum-free DMEM for further use.

### 2.4 Measurements of growth factors

The kinetics of PDGF-BB, VEGF, TGF-β, and fibroblast growth factor-2 (FGF-2) release from the prepared L-PRF were determined by enzyme-linked immunosorbent assay (ELISA) as previously described with a minor modification [31]. Briefly, 500 μL of L-PRF and F-PRF membrane fragments from each sample were immersed in 1 mL of PBS in 2-mL microcentrifuge tubes (n = 6). Then, all of the tubes were placed on a shaking table with continuous agitation at 37 °C. After 14 days of incubation, the supernatants were collected for analysis, and the same amount of fresh PBS was added to the tubes. The amounts of the aforementioned growth factors in the supernatants were determined using ELISA kits from R&D Systems (DB100; Minneapolis, MN) according to the manufacturer's protocols. The results were analyzed using an automatic enzyme labeling instrument at a wavelength of 450 nm. Subsequently, the bioactivity and stability of these factors in L-PRF and F-PRF were investigated in with respect to their ability to stimulate the proliferation of cultured BMSCs.

### 2.5 BMSC proliferation upon exposure to L-PRF extracts

The PRF extracts (medium containing PRF) used in this study refer to the PRF leaching solutions. Medium containing different concentrations of L-PRF or F-PRF (0×, 0.2×, 0.5×, and 1× L-PRF or F-PRF) were prepared as follows. L-PRF or F-PRF membranes were added to standard medium according to the indicated ratios for the release of autologous growth factors and were discarded after continuous release for 7 days. The concentration of PRF was calculated as follows: 1× PRF refers to PRF prepared from 1 mL of blood that was added to 1 of mL standard medium.

Cell proliferation was evaluated using a 3-[4,5-dimethyl(thiazol-2-y1)-3,5-diphenyl] tetrazolium bromide (MTT) (Sigma) assay. Briefly, BMSCs (200 μL of a 3×10^3^ cells/mL suspension in medium containing different concentrations of L-PRF or F-PRF) were seeded into the wells of a 96-well plate and incubated at 37 °C under a humidified atmosphere containing 5% CO_2_ to allow for cell attachment and spreading. Cell proliferation was analyzed every 24 h for 7 days using the MTT assay. Briefly, 20 μL of a 1 mg/mL MTT solution (Sigma-Aldrich) was added to each sample well and incubated for an additional 4 h at 37 °C. Subsequently, the reaction solution was removed and replaced with 150 μL of dimethyl sulfoxide (DMSO) for crystal solubilization, and the absorbance was then measured at 570 nm using a microplate reader (Beckman, Fullerton, CA). Each sample was assayed using five replicates.

### 2.6 Osteogenic capacity of BMSCs exposed to L-PRF

According to the results of the cell proliferation assay of BMSCs exposed to L-PRF, the optimal L-PRF concentration in osteogenic medium was determined to be 0.5× L-PRF, which was subsequently used in the experimental group to evaluate the osteogenic capacity of BMSCs exposed to L-PRF. The following four groups were investigated in this study: (1) the blank group (standard DMEM medium without osteoinductive supplements or PRF), (2) the control group (pure osteogenic medium without L-PRF or F-PRF), (3) the F-PRF group (osteogenic medium with 0.5× F-PRF), and (4) the L-PRF group (osteogenic medium with 0.5× L-PRF).

### 2.7 Alkaline phosphatase (ALP) activity

An ALP diagnostic kit (Sigma-Aldrich) was used to analyze the ALP activity of BMSCs according to the manufacturer's protocol and as previously described [22]. Briefly, BMSCs (seeded at a density of 5×10^4^ cells/cm^2^) were cultured in 12-well culture plates with osteogenic medium containing L-PRF or F-PRF, pure osteogenic medium, or standard medium. The cell sheets were harvested using a cell scraper, and cell lysates were obtained by washing the sheets with PBS followed by homogenization and sonication. The cell lysates (0.1 mL) were added to 0.5 mL of p-nitrophenol phosphate solution (Sigma-Aldrich) and 0.5 mL ALP buffer solution (Sigma-Aldrich). After incubating for 30 min at 37 °C, 10 mL of 0.05 N NaOH was added to stop the reaction, and a microplate reader (Bio-Rad, Hercules, CA) was used to measure the absorbance at 405 nm. ALP activity was normalized by cell numbers. ALP activity was expressed as the amount of p-nitrophenol (nM) released per million cells per minute. Six replicates were analyzed for each group, and the ALP measurements were performed in duplicate.

### 2.8 RNA isolation and quantitative RT-PCR

Total cellular RNA was isolated from BMSCs in the abovementioned groups and successively cultured for 14 days without passaging using an E.Z.N.A. Total RNA Kit (Omega Bio-Tek, Norcross, GA) according to the manufacturer's protocol. We evaluated the relative gene expression of the following osteogenic markers: collagen type I (COL I), osteopontin (OPN), osteocalcin (OCN), and bone morphogenetic protein 2 (BMP-2) using the corresponding primers shown in Table 1. The quality of the isolated RNA (2000 ng) was analyzed at 260/280 nm using a NanoDrop 2000/2000C spectrophotometer (Thermo Scientific, Waltham, MA). RNA was reverse-transcribed into cDNA using a PrimerScript™ RT Reagent Kit (Perfect Real Time) (TaKaRa Biotechnology, Dalian, China). The primers used for each of the target mRNA transcripts are shown in Table 1. SYBR® Premix Ex Taq™ (TaKaRa Biotechnology) was used to analyze the levels of target relative gene expression in an ABI 7500 Real-Time PCR Cycler (Applied Biosystems, Foster, CA) with the following thermocycling conditions: hot start at 95 °C for 30 s in the holding stage; 40 cycles of 95 °C for 15 s and 60 °C for 34 s in the cycling stage; and 95 °C for 15 s, 60 °C for 1 min, 95 °C for 30 s, and 60 °C for 15 s in the melting curve stage. The comparative threshold cycle (ΔΔCT) method was used to analyze the relative expression levels.

**Table 1:** Gene primer sequences used for RT-PCR.

Genes	Primer Sequences
*COL I*	F: 5′ GCCACTCTGAAGTCTCTGAACAAC 3′ R: 5′ TAGTAACCACTGCTCCACTCTGG 3′
*OPN*	F: 5′ CACTGAAGTCGTTCCCACAGTAG 3′ R: 5′ GTATCATCCAAGTCCTCGCTGTC 3′
*OCN*	F: 5′ TCTACCAGTTGCAGCCTGAC 3′ R: 5′ GTTCCCTTCCTCCTTGATTT 3′
*BMP-2*	F: 5′ CGTGAGGATTAGCAGGTCTTTG 3′ R: 5′ CGCTTGACGCTTTTCTCTTCTG 3′
*GAPDH*	F: 5′ GGGTGGTGGACCTCATGGT 3′ R: 5′ CGGTGGTTTGAGGGCTCTTA 3′

Note:COL I: collagen type I; OPN: osteopontin; OCN: osteocalcin; BMP-2: bone morphogenetic protein 2; GAPDH: glyceraldehyde-3-phosphate.

### 2.9 In vivo transplantation assay

To further investigate the effect of L-PRF on BMSC osteogenic capacity in vivo, a transplantation experiment was performed. Severe combined immunodeficiency (SCID) mice originally obtained from the animal center of SMU were bred and maintained under pathogen-free conditions. All procedures were reviewed and approved by the Animal Care Committee of SMU.

First, the L-PRF or F-PRF membrane fragments obtained from 10-mL blood samples were suspended in 0.5 mL of serum-free DMEM containing BMSC sheet fragments. Then, 24 injection sites on twelve 6-week-old male SCID mice weighing 20–25 g were randomly divided into the following three groups (n = 8 injection sites in each group): the control group (BMSC sheet fragments), the F-PRF group (BMSC sheet fragments and F-PRF membrane fragments) and the L-PRF group (BMSC sheet fragments and L-PRF membrane fragments). Shortly after the mice were anesthetized with 3% isoflurane gas, they were injected with 0.5 mL of BMSC sheet fragments (containing approximately 1.5×10^7^ BMSCs) or a mixture of BMSC sheet fragments and L-PRF or F-PRF membrane fragments into subcutaneous sites on the backs of the mice with an 18-gauge needle. The injections did not require skin incisions.

### 2.10 Microcomputed tomography (micro-CT) analysis

Eighty weeks after transplantation, all of the mice were euthanized with an overdose of anesthesia. A microcomputed tomography (micro-CT) system (Siemens Inveon Micro CT, Siemens AG, Munich, Germany) was used to image the backs of the SCID mice to evaluate changes in the tissue constructs before they were harvested for histological analysis. The samples were scanned at a resolution of 0.22 mm, with 692 scan slices obtained and reconstructed according to the manufacturer's references. The output was represented as three-dimensional stacks using Inveon Research Workplace (Siemens AG). Based on threshold measurements for samples of porcine femur bone, the thresholds used in this study were 68–1732 Hounsfield Units (HU) for cortical bone and -70-67 HU for cancellous bone. The ratio of the bone volume/total volume (BV/TV) was also measured.

### 2.11 Histological analysis of the transplanted constructs

After micro-CT scanning, the newly formed masses were harvested and observed macroscopically. The specimens were then fixed in 4% paraformaldehyde for 24 h and divided into two halves, one of which was decalcified for 2 weeks in 10% ethylenediaminetetraacetic acid (EDTA) at pH 8.0. The specimens were then embedded in paraffin, after which the paraffin blocks were sectioned into 4-μm thick slices, stained with hematoxylin and eosin (H&E), and then observed by light microscopy (DX51, Olympus, Tokyo, Japan).

The other half of each specimen was fixed in 10% formaldehyde for 1 week at room temperature. Subsequently, the samples were dehydrated in an ascending graded series of ethanol (70–100%) and then saturated and embedded in methyl methacrylate. Serial sections (150 μm) were prepared using a high-speed precision microtome (Leica SP1600, Wetzlar, Germany). All of the sections were ground to a thickness of 35 μm, stained with Masson's trichrome (MTC) and observed by light microscopy.

Sections were selected from each specimen for histomorphometrical examination as reported previously. After performing Masson's trichrome staining, the samples were examined using a light microscope (Leica Microsystems AG, Wetzlar, Germany). Two unbiased examiners who were blinded to the experimental factors randomly observed and recorded three high-resolution low-magnification images of each section and then analyzed these images twice using computer-based image analysis techniques (Leica Qwin Pro-Image Analysis System). The cross-sectional area of the newly formed mineralized bone and cartilage (blue staining) was calculated and reported as the percentage of the stained area relative to the entire cross-sectional area.

### 2.12 Statistical analysis

Data were collected from at least triplicate parallel samples in vitro and from eight samples in vivo and presented as the means ± standard deviation (SD). Statistical analysis was performed using SPSS 17.0 (SPSS, Chicago, IL, USA). Analysis of variance (ANOVA) was used for multiple group comparisons followed by Tukey's honestly significant difference test, with P < 0.05 considered to indicate significance.

## 3 Results

### 3.1 Characterization of lyophilized PRF (L-PRF) and fresh PRF (F-PRF)

**Fig. 1:**
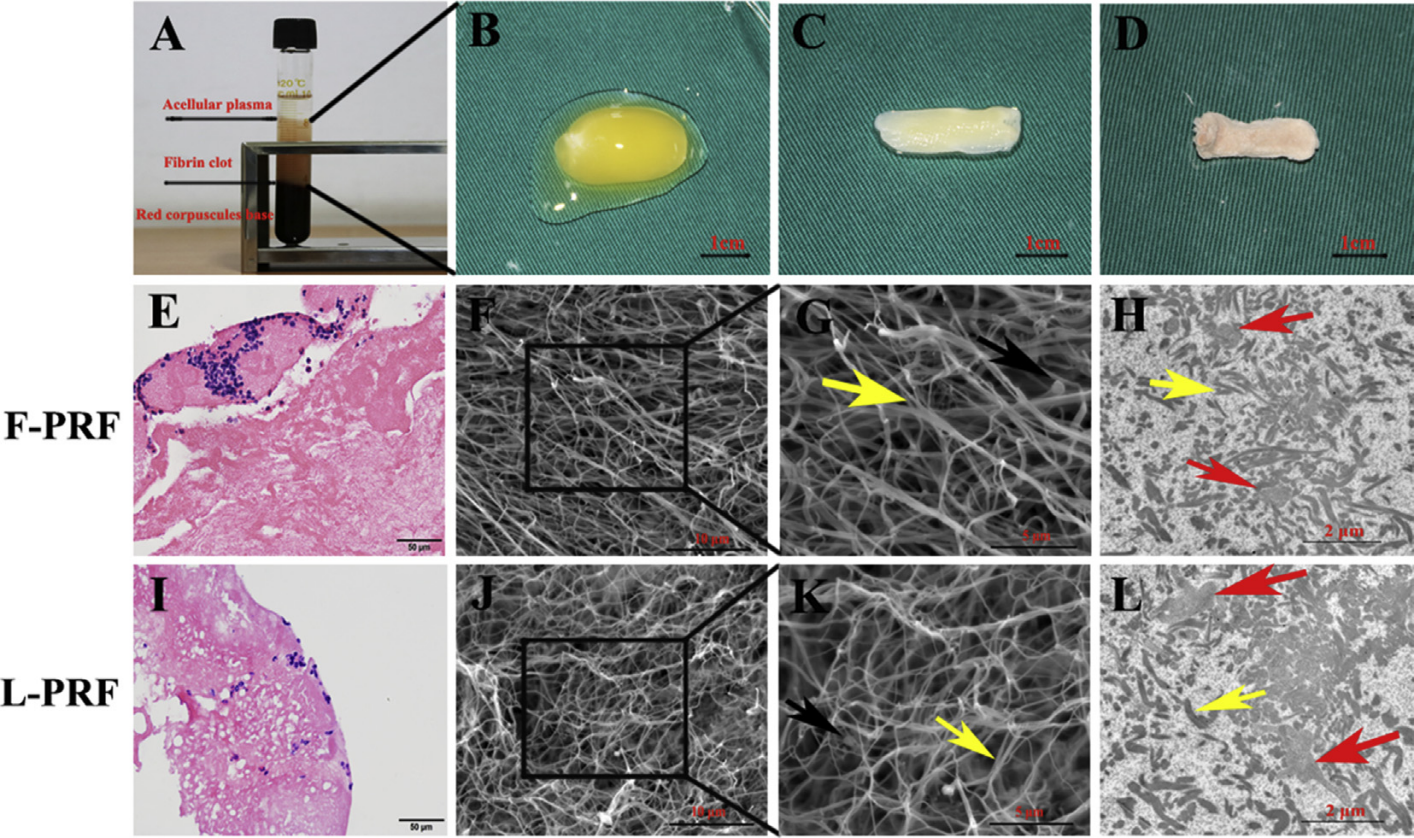
Characterization of L-PRF and F-PRF. (A) Blood samples were divided into three layers after centrifugation for 10 min at 3000 rpm. The top layer of pale yellow liquid was acellular plasma, the bottom layer of loose red jelly was the red blood cell layer, and the pale yellow gel in the middle contained fibrin clots. (B) A PRF clot was easily obtained when the acellular plasma and red blood cells were removed (scale bar = 1 cm). (C) A compact, flexible and strongly elastic fibrin membrane, which was considered to be F-PRF, was obtained by removing the fluids trapped within the fibrin matrix (scale bar = 1 cm). (D) After lyophilization, L-PRF with a rough surface and loose structure was collected (scale bar = 1 cm). (E) H&E staining revealed that F-PRF consisted of a large number of closely arranged red-stained fiber bundles containing numerous blue-stained nuclear leukocytes (scale bar = 50 μm). (I) The fibrin network of L-PRF was loosely arranged with numerous pores containing many well-distributed blue-stained nuclear leukocytes (scale bar = 50 μm). (F, J) SEM examination showed that fibrin was arranged regularly in the F-PRF (F), whereas the arrangement of fibrin in the L-PRF was disordered (J) (scale bar = 10 μm). (G, K) High-magnification SEM images showed the three-dimensional network of PRF, which consisted of a large number of trimolecular branch junctions (yellow arrow, G) with fibrin fibers and some fibrillae with a smaller diameter (yellow arrow, K) in both F-PRF (G) and L-PRF (K). Among the fibrin fibers, a few platelets were also observed (black arrow, scale bar = 5 μm). (H, L) TEM examination also demonstrated that both F-PRF (H) and L-PRF (L) consisted of numerous fibrin bundles (yellow arrow). The α-granules of platelets in the F-PRF (H) were maintained intact, and each platelet was tightly adhered to several fibrin bundles (red arrow, H). However, some α-granules of the platelets in the L-PRF were damaged, and part of the platelet membrane structure vanished (red arrow, L). The platelets were slightly larger and swollen and staining was very weak in the L-PRF (L) compared with the F-PRF (H) (scale bar = 2 μm). These results suggested that freeze-drying had little effect on the structure of PRF.

Ten milliliters of whole blood was divided into three layers after the samples were collected and immediately centrifuged for 10 min at 3000 rpm. The top layer of pale yellow liquid was acellular plasma, the bottom layer of loose red jelly was the red blood cell layer, and the pale yellow gel in the middle consisted of fibrin clots (Fig. 1A). PRF clots were easily obtained when the acellular plasma and red blood cells were removed (Fig. 1B). A compact, flexible and strongly elastic fibrin membrane, which was considered to be F-PRF, was obtained by removing the fluids trapped within the fibrin matrix (Fig. 1C). After lyophilizing the F-PRF, L-PRF with a rough surface and loose structure was collected (Fig. 1D).

The histological observations showed that F-PRF consisted of a large number of closely arranged, red-stained fiber bundles containing numerous blue-stained mononuclear leukocytes (Fig. 1E). However, the L-PRF fibrin network was loosely arranged with numerous pores that contained numerous distributed blue-stained nuclear leukocytes (Fig. 1I). SEM examination showed that fibrin was arranged regularly in the F-PRF, whereas the arrangement of fibrin was disordered in the L-PRF (Fig. 1F, J). However, the three-dimensional network consisted of a large number of trimolecular branch junctions (yellow arrow, Fig. 1G) with fibrin fibers, and some fibrillae with a smaller diameter (yellow arrow, Fig. 1K) were observed in both the F-PRF and L-PRF. Among the fibrin fibers, a few platelets were also observed (black arrow, Fig. 1G, K). TEM examination also demonstrated that both the F-PRF and L-PRF consisted of numerous fibrin bundles (yellow arrow, Fig. 1H, L). The α-granules of platelets in the F-PRF were maintained intact, with each platelet tightly adhered to several fibrin bundles (red arrow, Fig. 1H). However, some α-granules of the platelets in the L-PRF were damaged, and part of the platelet membrane structure vanished (red arrow, Fig. 1L). In addition, the platelets were slightly larger and swollen, and the staining was very weak in the L-PRF compared with the F-PRF (Fig. 1H, L). These results suggested that freeze-drying had little effect on the PRF structure.

### 3.2 Characterization of BMSC sheets

**Fig. 2:**
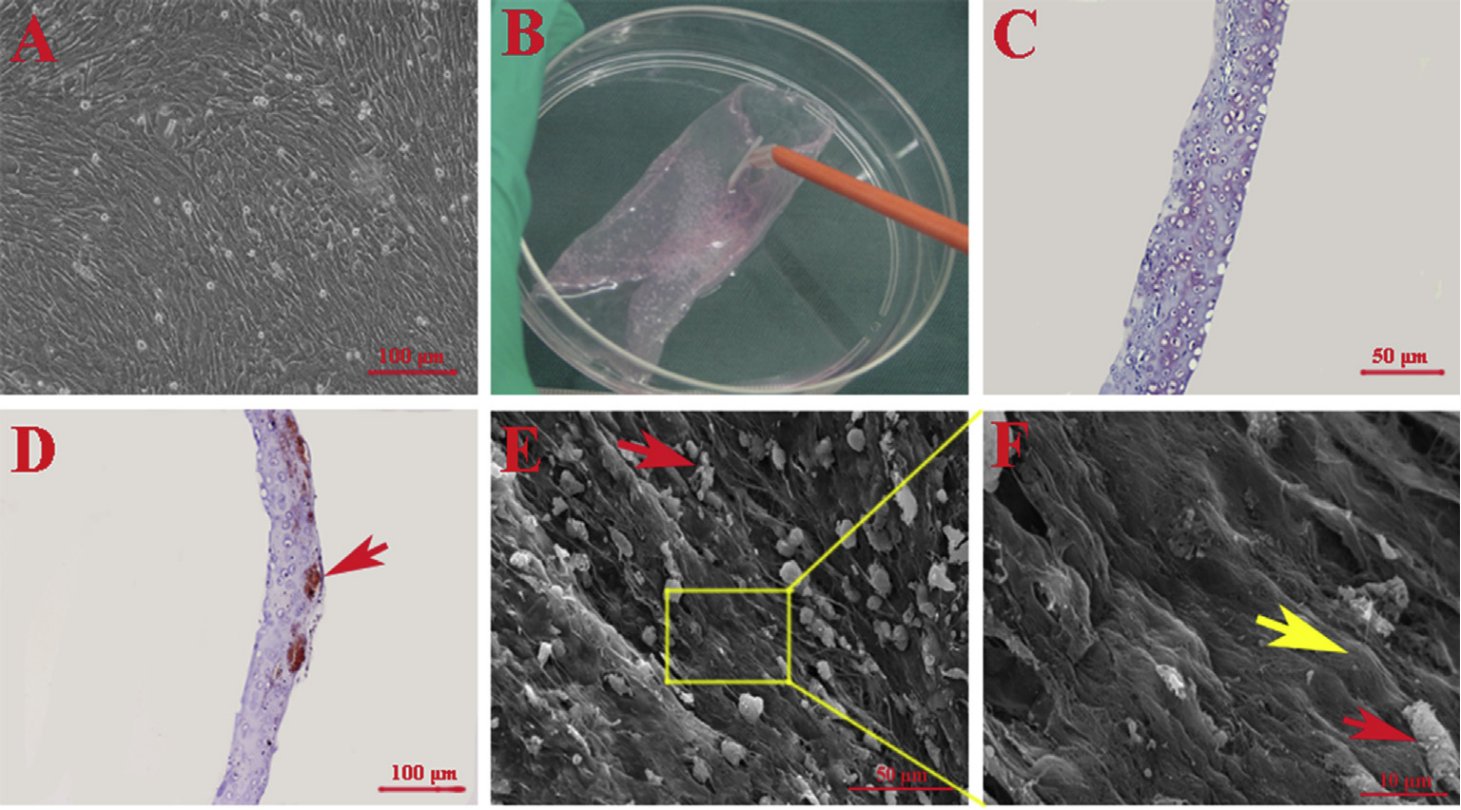
Characterization of BMSC sheets. (A) The cultured rabbit cells had a fibroblast-like morphology and grew well with a close arrangement (scale bar = 100 μm). (B) After the BMSCs were continuously cultured in osteogenic medium for 2 weeks, multiple layers of BMSCs formed a thick cell sheet that was easily lifted from the culture dish with a cell scraper and maintained intact. (C) Histological analysis demonstrated that the obtained sheet consisted of several layers of cells that secreted autologous ECM (scale bar = 50 μm). (D) Alizarin red staining further verified that calcium deposits (red arrow) formed throughout the cell sheet (scale bar = 100 μm). (E) SEM analysis also showed obvious mineral-like nodules (red arrow) on the surface of the cell sheets (scale bar = 50 μm). (F) High-magnification SEM images showed that BMSCs were trapped and embedded within autologous secreted ECM (yellow arrow); the red arrow indicates mineral-like nodules (scale bar = 10 μm).

The cultured rabbit cells displayed good stem cell characteristics, including self-renewal and rapid proliferation. The BMSCs had a fibroblast-like cell morphology and grew well with a close arrangement (Fig. 2A). After BMSCs were initially seeded at a density of 5×10^4^ cells/cm^2^ and continuously cultured in osteogenic medium without passaging for 14 days, multiple layers of BMSCs formed a thick cell sheet that was easily detached from the culture dish with a cell scraper and maintained intact (Fig. 2B). Histological analysis demonstrated that the obtained sheet consisted of several layers of cells that secreted autologous ECM (Fig. 2C). Moreover, alizarin red staining further verified that calcium deposits formed throughout the cell sheet (red arrow, Fig. 2D). High-magnification SEM analysis also showed obvious mineral-like nodules on the surface of the cell sheets (red arrow, Fig. 2E, F), and BMSCs were trapped and embedded in autologous secreted ECM (yellow arrow, Fig. 2F).

### 3.3 Biological characteristics of F-PRF and L-PRF

**Fig. 3:**
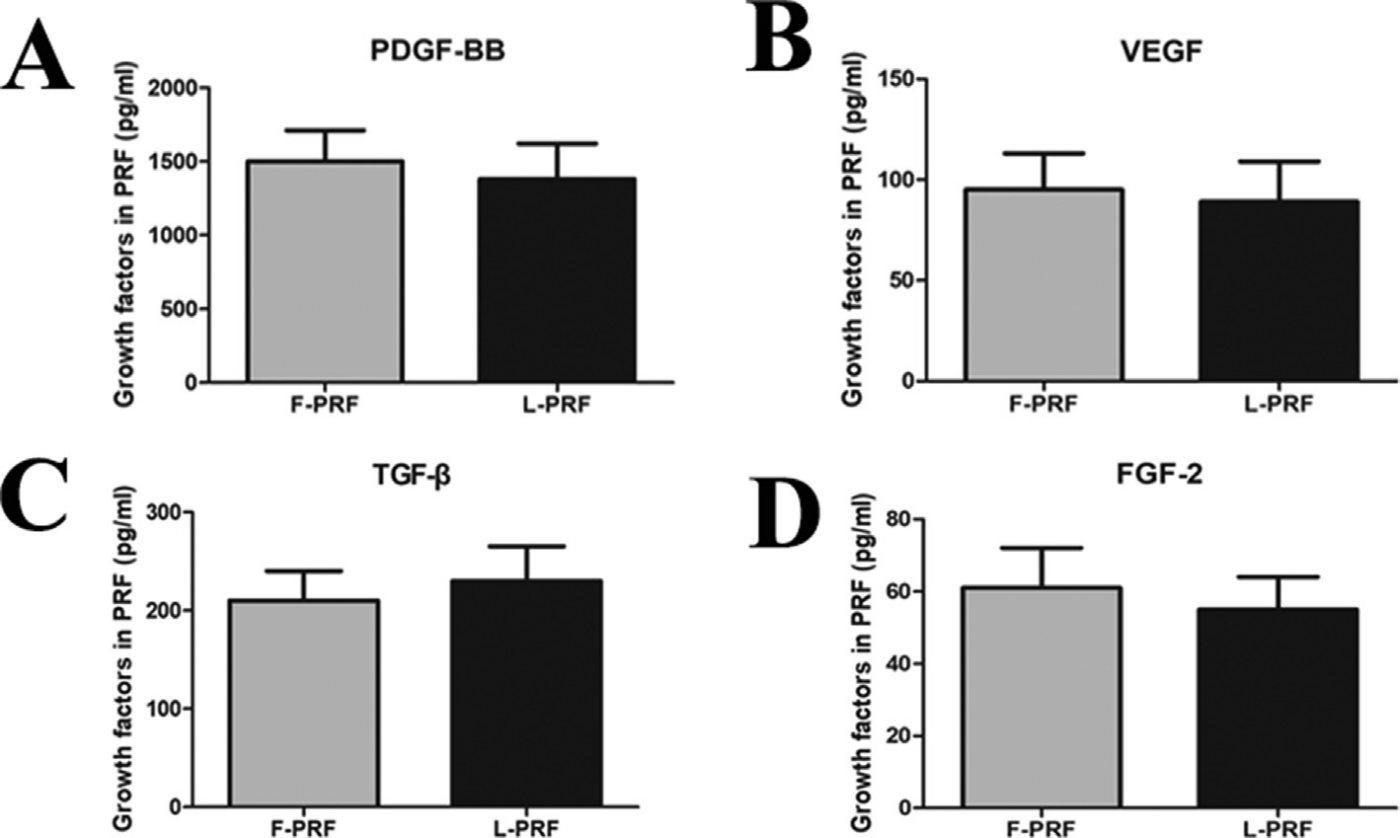
Biological characteristics of F-PRF and L-PRF. (A–D) A comparison of the F-PRF- and L-PRF-associated growth factors. The levels of the growth factors PDGF-BB (A), VEGF (B), TGF-β (C) and FGF-2 (D) were analyzed by F-PRF- and L-PRF-associated. No significant differences were observed in the levels of these growth factors between the F-PRF and L-PRF samples (P = 0.13).

The F-PRF and L-PRF supernatants were transferred to 96-well plates at a proportion of 5%, and the levels of the growth factors (PDGF-BB, VEGF, TGF-β and FGF-2) were measured by ELISA. As indicated by the ELISA results, 500 μL of F-PRF contained 1500.2 ± 210.4 pg/mL PDGF-BB, 95.3 ± 18.5 pg/mL VEGF, 210.5 ± 30.8 pg/mL TGF-β, and 61.4 ± 11.3 pg/mL FGF-2. In addition, 500 μL of L-PRF contained 1380.9 ± 240.1 pg/mL PDGF-BB, 89.6 ± 20.3 pg/mL VEGF, 230.1 ± 35.2 pg/mL TGF-β, and 55.7 ± 9.2 pg/mL FGF-2 (Fig. 3A–D). There were no significant differences in the levels of the assayed growth factors between the F-PRF and L-PRF samples (P = 0.13) (Fig. 3A–D).

**Fig. 4:**
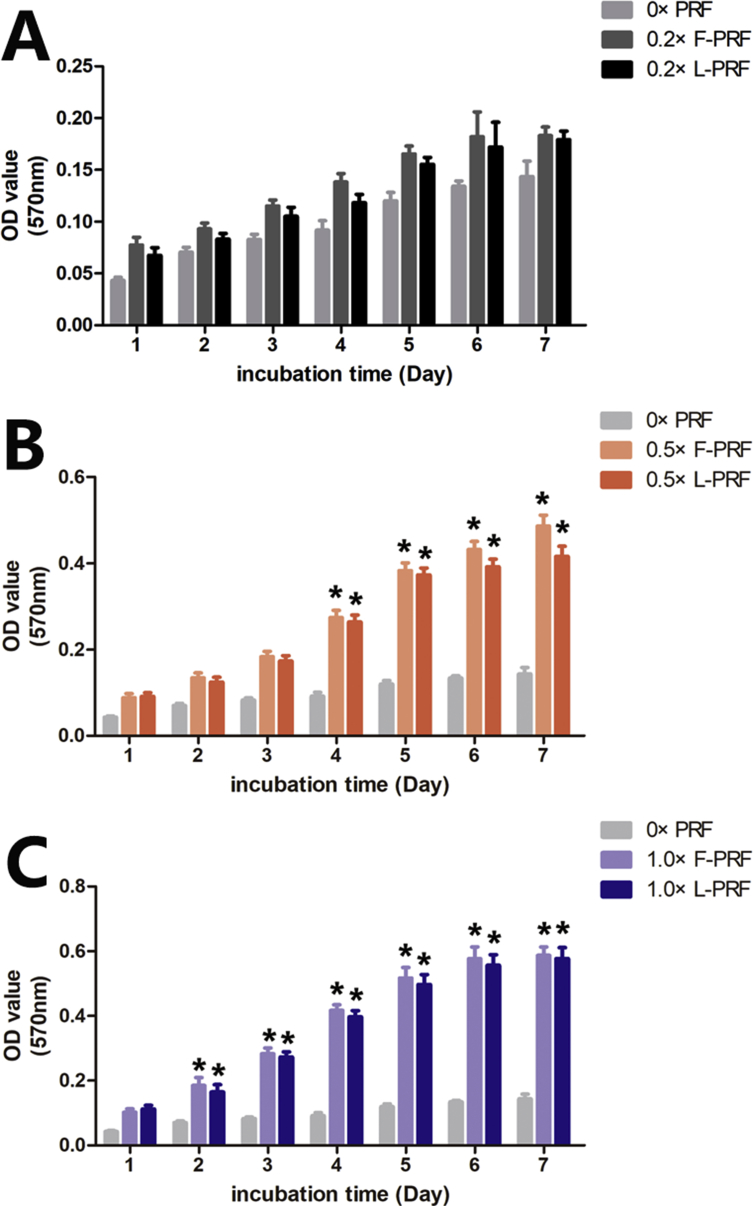
The effects of L-PRF on the proliferation capacity of BMSCs. MTT assays were performed to examine the effects of L-PRF on the proliferation of BMSCs. The proliferative ability of BMSCs significantly improved by increasing the L-PRF or F-PRF concentrations from 0.2× PRF to 1× PRF in the culture medium. (A–C) Enhanced cell proliferation was observed in the 0.5× and 1× PRF groups compared with that observed in the control and 0.2× PRF groups on the fourth day of cultivation (P = 0.01). Moreover, enhanced cell proliferation was observed in the 1× PRF groups compared with the 0.5× PRF groups starting on the third day of cultivation (P = 0.01). However, a significant difference between the F-PRF and L-PRF groups at the same time point was not observed (P > 0.05). These results showed that L-PRF had a significant, dose-dependent and durable biological stimulative effect on the proliferative capacity of BMSCs in vitro. * Indicates a significant difference compared to the control group (P < 0.05).

MTT assays were performed to examine the effects of L-PRF on the proliferation of BMSCs. As shown in Fig. 4, the proliferative ability of BMSCs significantly improved by increasing the L-PRF or F-PRF concentrations in the culture medium from 0.2× PRF to 1× PRF. Enhanced cell proliferation was observed in the 0.5× and 1× PRF groups compared with the control and 0.2× PRF groups on the fourth day of cultivation (P = 0.01). Moreover, enhanced cell proliferation was observed in the 1× PRF groups compared with the 0.5× PRF groups starting on the third day of cultivation (P = 0.01). However, a significant difference between the F-PRF and L-PRF groups at the same time point was not observed (P = 0.09). These results showed that L-PRF had a significant, dose-dependent and durable biological stimulative effect on the proliferative capacity of BMSCs in vitro.

### 3.4 Effects of L-PRF on the osteogenic capacity of BMSC sheets

**Fig. 5:**
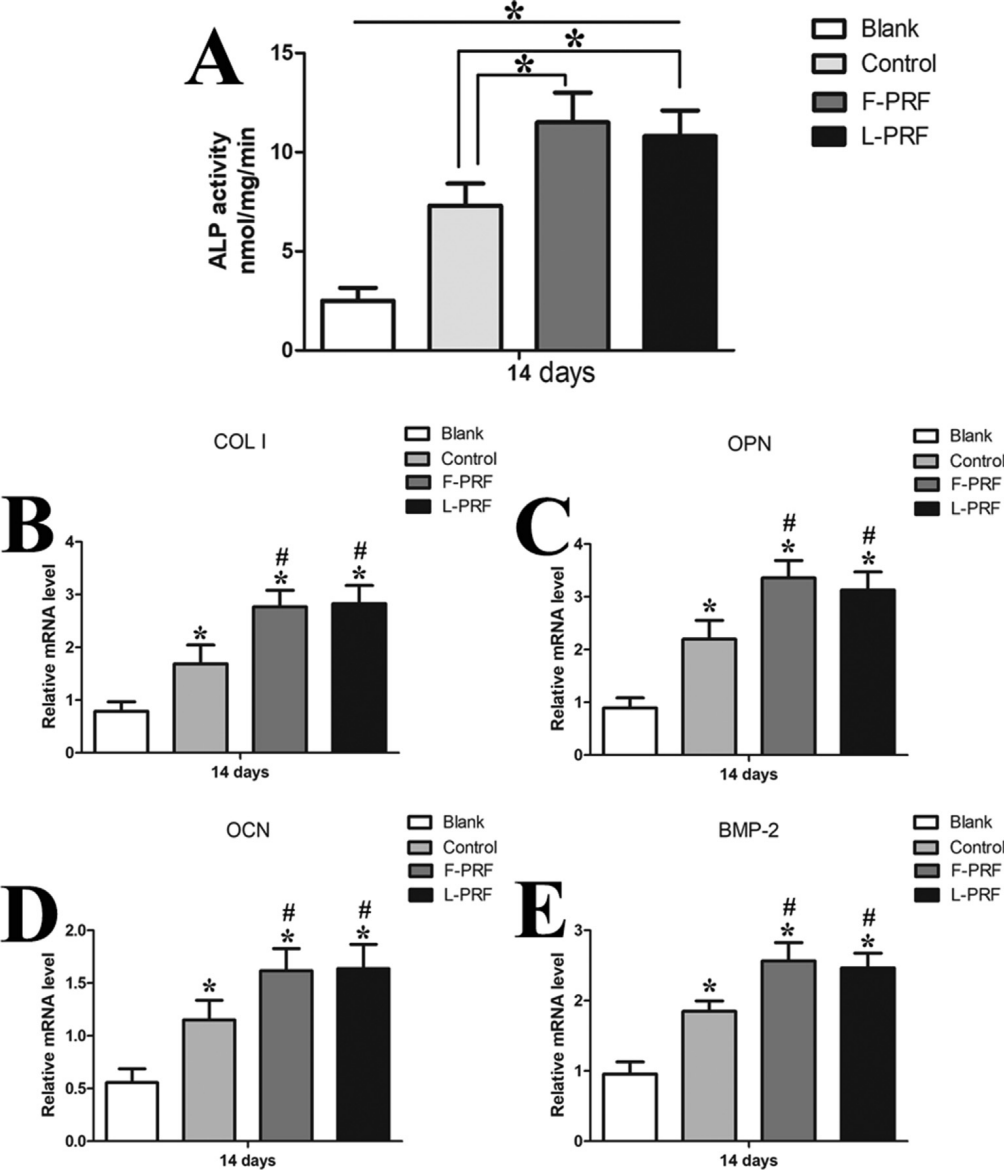
The effects of L-PRF on the osteogenic capacity of BMSC sheets. (A) The ALP activity in the blank group was significantly less than that that of the control and PRF groups at day 14 (P = 0.01). Moreover, the ALP activity in the L-PRF and F-PRF groups was notably greater than that observed in the control group (P < 0.05). However, there was no significant difference in the ALP activity between the F-PRF and L-PRF groups (P = 0.15). (B–E) Gene expression was analyzed by RT-PCR. Among all the groups, the blank control had the lowest relative gene expression levels for COL I, OPN, OCN, and BMP-2, which were significantly less than those observed in the control and PRF groups (P = 0.01). The relative gene expression levels of COL I, OPN, OCN, and BMP-2 were all highly upregulated in the L-PRF and F-PRF groups compared with that observed in the control group (P = 0.01). There were no significant differences in the relative levels of gene expression for these osteogenic markers between the F-PRF and L-PRF groups (P > 0.05). * Indicates a significant difference compared to the blank group (P < 0.05). # Indicates a significant difference compared to the control group (P < 0.05).

The ALP activity in the blank group was significantly less than that observed in the control and PRF groups at day 14 (P = 0.01) (Fig. 5A). Moreover, the ALP activity in the L-PRF and F-PRF groups was notably greater than that observed in the control group (P = 0.01). However, there was no significant difference in the ALP activity between the F-PRF and L-PRF groups (P = 0.15) (Fig. 5A).

Among all of the groups, the blank control group had the lowest relative gene expression levels for COL I, OPN, OCN, and BMP-2, which were significantly lower than those observed in the control and PRF groups (P = 0.01) (Fig. 5B–E). The relative gene expression levels of COL I, OPN, OCN, and BMP-2 were all highly upregulated in the L-PRF and F-PRF groups compared with those observed in the control group (P = 0.01) (Fig. 5B–E). There were no significant differences in the relative gene expression levels of these osteogenic markers between the F-PRF and L-PRF groups (P = 0.13) (Fig. 5B–E). Thus, the induced BMSC sheets in the L-PRF group had a higher osteogenic differentiation potential than those in the blank and control groups in vitro, indicating that L-PRF significantly improved the osteogenic differentiation of BMSC sheets in vitro.

### 3.5 Gross morphology and microcomputed tomography (micro-CT) analysis of bone tissue formed in vivo

**Fig. 6:**
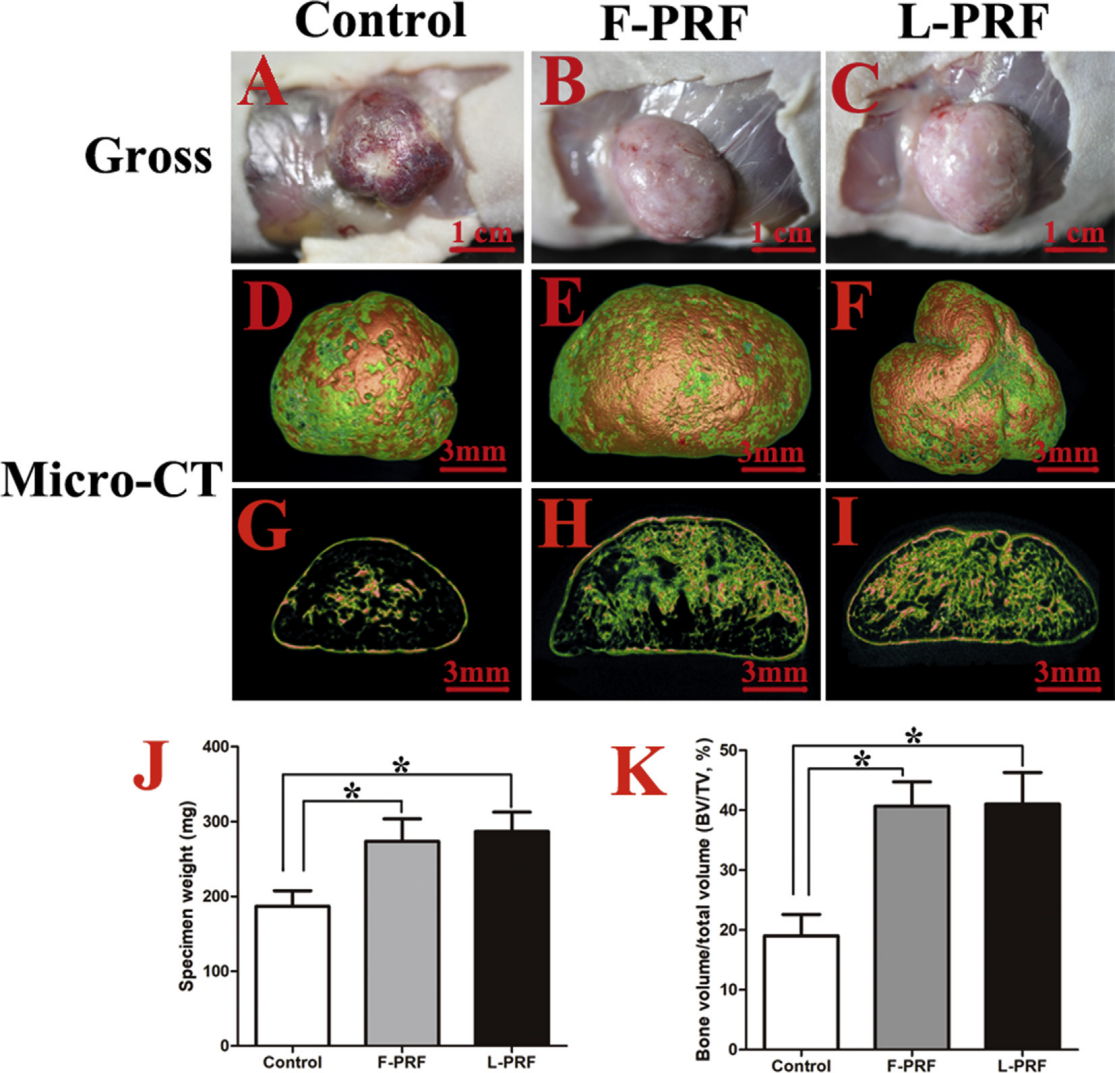
Gross morphology and micro-CT analysis of bone tissue formed in vivo. (A–C) The harvested specimens in the PRF groups were harder than those from the control group (scale bar = 1 cm). (D–F) The micro-CT images showed a mass of deposited minerals in all of the groups, but the spongy bone structure in the control group (D) was much sparser than that observed in the F-PRF (E) and L-PRF (F) groups (scale bar = 3 mm). (G–I) Cross sections of the micro-CT images also showed that there was substantially more newly formed bone-like tissue in the F-PRF (H) and L-PRF (I) groups than in the control group (G) (scale bar = 3 mm). (J) A comparison of specimen weights between the groups. The average weight of the specimens in the control group was 186.7 ± 22.5 mg, which was significantly less than that observed in the F-PRF (273.3 ± 31.6 mg) and L-PRF (286.7 ± 29.5 mg) groups (P = 0.01), with no significant difference observed between the F-PRF and L-PRF groups (P = 0.14). (K) The three-dimensional bone volume to total volume (BV/TV) ratios indicated significant differences in the amount of newly formed bone between the control and PRF groups (P = 0.01). Specifically, the BV/TV in the F-PRF and L-PRF groups was 40.3 ± 5.5 and 41.4 ± 6.8%, respectively, which was notably greater than that observed in the control group (19.1 ± 2.5%) (*P < 0.05).

Eight weeks after implantation, ectopic bone-like tissues formed in the control and PRF groups exhibited no obvious inflammation, and the newly formed oval-shaped tissue in all specimens was intact. The gross investigation indicated that the harvested specimens in the PRF groups were harder than those of the control group (Fig. 6A–C). The average weight of the specimens in the control group was 186.7 ± 22.5 mg, significantly less than that of the F-PRF (273.3 ± 31.6 mg) and L-PRF (286.7 ± 29.5 mg) groups (P = 0.01) (Fig. 6J), with significant difference observed between the F-PRF and L-PRF groups (P = 0.14) (Fig. 6J). The micro-CT images showed a mass of deposited minerals in all of the groups, but the spongy bone structure in the control group (Fig. 6D) was more porous than that observed in the PRF groups (Fig. 6E, F). Cross sections of the micro-CT images also showed that there was substantially more newly formed bone-like tissue in the PRF groups (Fig. 6E, F) than in the control group (Fig. 6G). Additionally, the three-dimensional BV/TV ratios measured from the micro-CT images indicated that there were significant differences in the amount of newly formed bone between the control and PRF groups (P = 0.01) (Fig. 6K). Specifically, the BV/TV values in the F-PRF and L-PRF groups were 40.3 ± 5.5 and 41.4 ± 6.8%, respectively, notably greater than that observed in the control group (19.1 ± 2.5%) (P = 0.01) (Fig. 6K).

### 3.6 Histological analysis of newly formed bone tissue

**Fig. 7:**
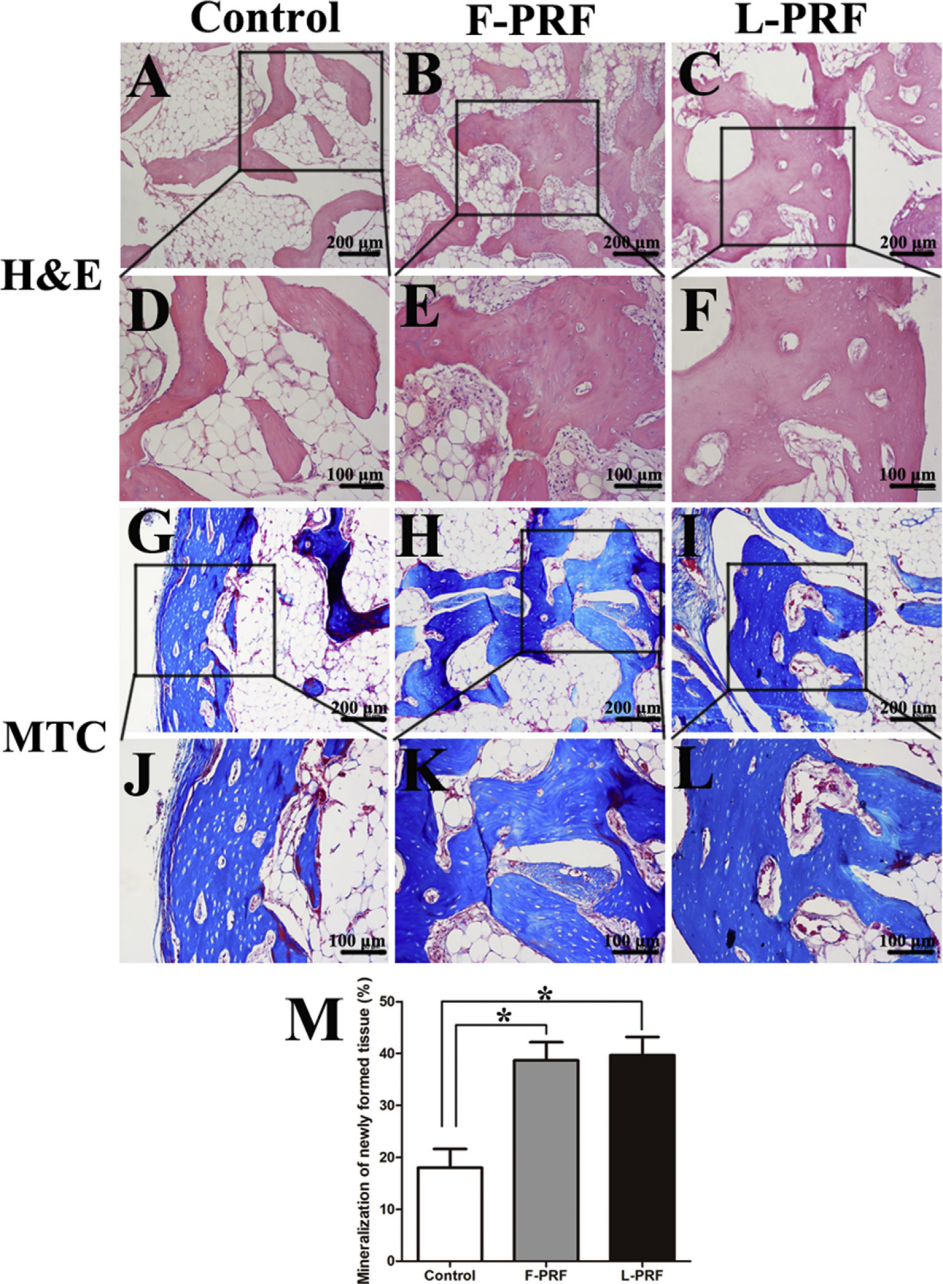
Histological analysis of newly formed bone tissue. HE staining. (A) Compared with the PRF groups, the control group showed fewer newly formed island-like bone tissue structures (scale bar = 200 μm). (B, C) A large number of interconnected ossified trabeculae and woven bone structures were observed in the F-PRF (B) and L-PRF (C) groups (scale bar = 200 μm). (D) A high-magnification image indicated that the newly formed bone and cartilage-like tissues were sparse and unevenly distributed in the control group (scale bar = 100 μm). (E, F) Osteoblasts were observed in the newly mineralized tissue, osteocytes were embedded in the dense matrix, and primary bone marrow was observed in the F-PRF (E) and L-PRF (F) groups (scale bar = 100 μm). (C–L) Masson's trichrome staining showed that the F-PRF (H, K) and L-PRF (I, L) groups exhibited more osteogenesis than the control group (G, J) (scale bar = 200 μm in G, H, and I, and scale bar = 100 μm in J, K, and L). (M) Histomorphometrical examination of the specimens indicated that mineralized bone and cartilage occupied 38.7 ± 3.9 and 39.6 ± 4.2% of the total cross-sectional areas of the specimens in the F-PRF and L-PRF groups, respectively (P = 0.09). However, the percent area of new bone and cartilage in the control group (18.2 ± 2.3%) was significantly less than that observed in the F-PRF and L-PRF groups at eight weeks postimplantation (P = 0.01) (*P < 0.05).

Twenty-four specimens were assessed by light microscopy after H&E and Masson's trichrome staining. Both osteogenesis and angiogenesis were observed within the groups, but notable differences were observed in the newly formed bone between the control and PRF groups. Fewer newly formed island-like bone tissue structures were observed in the control group compared with the PRF groups, and the newly formed bone and cartilage-like tissues were sparse and unevenly distributed (Fig. 7A, D). In contrast, a large number of interconnected ossified trabeculae and woven bone structures were observed in the F-PRF and L-PRF groups (Fig. 7B, C, E, F). In addition, osteoblasts were observed in the newly mineralized tissue, and osteocytes were embedded in the dense matrix (Fig. 7E, F). Histomorphometrical examination of the specimens indicated that mineralized bone and cartilage occupied 38.7 ± 3.9 and 39.6 ± 4.2% of the total cross-sectional areas of the specimens in the F-PRF and L-PRF groups, respectively (P = 0.09) (Fig. 7G–M). However, the percent area of new bone and cartilage in the control group (18.2 ± 2.3%) (Fig. 7G, J) was significantly less than that observed in the F-PRF and L-PRF groups at eight weeks postimplantation (P = 0.01) (Fig. 7M).

## 4 Discussion

In this study, we first compared the microstructure of L-PRF and F-PRF membranes and demonstrated that the L-PRF consisted of numerous densely arranged fibrin fibers and released growth factors. Next, we investigated the effects of L-PRF on BMSC proliferation and the expression of osteogenesis-related genes in vitro. Finally, we assessed bone formation when fragments of BMSC sheets with L-PRF or F-PRF membranes were ectopically transplanted via injection into SCID mice.

Because PRF gradually releases autologous growth factors during a slow degradation process and has a good ability to stimulate tissue regeneration, PRF has been widely used in the repair of bone and cartilage defects and in plastic surgery and has achieved satisfactory therapeutic results [19, 21]. However, the primary limitation of PRF is that since it is commonly used immediately after preparation in autologous applications, the long-term storage and commercialization of PRF is difficult. In this study, we prepared L-PRF by lyophilizing F-PRF in a vacuum freeze dryer and investigated the effect of L-PRF on the osteogenic potential of BMSCs in vitro and in vivo. Our results suggested that L-PRF consists of numerous densely arranged fibrin fibers and releases growth factors. L-PRF significantly stimulates the proliferative and osteogenic capacity of BMSCs in vitro. Furthermore, L-PRF and osteoblastic BMSC sheet fragments can be combined for bone tissue engineering in vivo. Considering that minimally invasive surgeries are becoming more popular in the clinic, the injection of fragments of BMSC sheets and L-PRF is simple and enables rapid rehabilitation, promoting the therapeutic effect of bone tissue engineering [13, 22]. The advantages of PRF include its high efficiency, low cost, and a simple preparation procedure without the need for additional chemical agents [32]. To address the storage issues and extend the clinical applications of PRF, such as the allogenic use of F-PRF, in this study, we investigated the procedure F-PRF and the applications of PRF. The freeze-drying process, which has been used for platelet preservation, is a common method used to improve the stability of tissue-regenerated proteins and facilitate their long-term preservation [30]. F-PRF was first frozen overnight in a cryorefrigerator at -80 °C and then quickly transferred to a vacuum freeze dryer for lyophilization. The histological examination showed that L-PRF derived from freeze-dried F-PRF had more loose and porous structures than F-PRF (Fig. 1E, I), similar to previously published results [22, 33]. Ultramicrostructural examination by SEM also verified that the arrangement of fibrin was disordered and sparse in L-PRF, whereas fibrin was arranged regularly in F-PRF (Fig. 1F, J). This large three-dimensional network structure of L-PRF can facilitate cell migration and tissue bonding [34]. Moreover, the freeze-drying method not only promotes cell proliferation and mineralization by increasing the pore diameter of L-PRF but also accelerates the degradation rate of L-PRF, which was greater than that of F-PRF, by increasing the liquid contact areas of L-PRF [35]. Moreover, as shown in Fig. 1, significantly fewer leukocytes were observed in L-PRF (Fig. 1I) compared to that observed F-PRF (Fig. 1E), indicating that the freeze-drying treatment may further reduce the number of PRF-associated leukocytes and potentially decreasing its immunological rejection.

PRF, which has potential as a new biomaterial in regenerative medicine, provides an abundance of autologous cytokines and growth factors [19, 36]. For example, F-PRF has been shown to markedly stimulate BMSC proliferation and differentiation in a dose-dependent manner in vitro [22]. In addition, F-PRF recruits stem cells and progenitor cells to injured sites and then triggers the tissue regeneration process by promoting the rapid proliferation and differentiation of MSCs [23, 25]. By measuring the PDGF-BB, VEGF, TGF-β, and FGF-2 contents released from L-PRF by ELISA, we observed that there were no significant differences in the levels of these growth factors between F-PRF and L-PRF (P > 0.05) (Fig. 3A–D). The MTT assays also showed that L-PRF had a significant, dose-dependent and durable biological stimulative effect on the proliferative potential of BMSCs in vitro (Fig. 4). Furthermore, the ALP activity assays and reverse transcription polymerase chain reaction (RT-PCR) results also confirmed that similar to F-PRF, L-PRF improved the osteogenic potential of BMSCs in vitro (P = 0.13) (Fig. 5A–E). These results indicated that L-PRF is a novel biomaterial scaffold that is suitable for the sustained release of growth factors and the protection of fibrogenic factors that are essential for protecting engineered tissue against proteolytic degradation [37, 38]. In addition, F-PRF has been demonstrated to stimulate the healing of articular cartilage defects in vivo [39].

After BSMCs are seeded and continuously cultured without passaging for two weeks, they expand approximately 3–4 times and secrete ECM that can be mechanically handled. Therefore, the expanded cells with intact cell-cell connections and their endogenously produced ECM can be harvested as a cell sheet [13, 40]. Because of the three-dimensional architecture and composition of ECM, it has a positive effect on cell chemotaxis, guides cell differentiation and induces beneficial host tissue remodeling [41]. Therefore, the cell sheet technique has been widely used in the regeneration of different types of tissues, such as bone, cartilage, fat, skin, liver, bladder, and heart tissues [12, 22, 40, 42]. In this study, we observed that L-PRF significantly improved the osteogenic potential of BMSC sheets in vivo by a gross morphological investigation and micro-CT and histological examinations, with no significant differences observed between the F-PRF and L-PRF groups. There are several advantages of the approach described in this study compared with other reported methods for fabricating tissue-engineered bone. First, this approach avoids the limitations associated with scaffold transplantation, as it does not require an exogenous scaffold [13]. Second, L-PRF, which possesses satisfactory biocompatibility and biodegradability, is used as a scaffold and as a source of growth factors to fabricate engineered bone [22]. Finally, the combined injection of L-PRF and osteogenic BMSC sheet fragments for bone tissue engineering is minimally invasive, allowing for a more rapid rehabilitation and extraordinarily decreases potential scar formation [43]. In addition, L-PRF can be prepared as different types of solid materials, such as sheets, granules and powders, according to clinical needs. L-PRF can also be used to prepare wound excipients or can be mixed with other materials for tissue engineering. However, there were some limitations of our study, and additional problems need to be solved. For example, we used fetal bovine serum in the cell culture, which may have influenced the obtained results. In addition, we did not investigate the associated molecular mechanisms, which are of particular interest and is a key issue that needs to be explored in the future.

## 5 Conclusion

In summary, the results of this study demonstrated that L-PRF derived from lyophilized F-PRF consists of numerous densely arranged fibrin fibers, releases growth factors and favors BMSC proliferation and osteogenic differentiation in vitro. The combined use of L-PRF and osteogenic BMSC sheet fragments enabled bone tissue regeneration when they were ectopically transplanted into SCID mice via injection. Overall, our results indicated that L-PRF increases the osteogenic potential of BMSCs in vitro and in vivo. Our data suggest that the use of BMSCs as cell sheets is effective, and BMSC sheets combined with L-PRF enriched with fibrin and growth factors may provide insights into the fabrication of engineered bone. However, additional studies on the molecular mechanisms of BMSC sheets and L-PRF interactions need to be conducted before this approach can be considered for use in patients with bone defects or osteonecrosis.

## Declarations

### Author contribution statement

Zhifa Wang: Conceived and designed the experiments; Performed the experiments; Analyzed and interpreted the data; Contributed reagents, materials, analysis tools or data; Wrote the paper.

Leng Han: Performed the experiments; Analyzed and interpreted the data; Contributed reagents, materials, analysis tools or data.

Tianyu Sun: Performed the experiments; Analyzed and interpreted the data.

Weijian Wang, Xiao Li: Analyzed and interpreted the data; Contributed reagents, materials, analysis tools or data.

Buling Wu: Conceived and designed the experiments; Analyzed and interpreted the data; Wrote the paper.

### Funding statement

This work was supported by the National Natural Science Foundation of China (81700943), the China Postdoctoral Science Foundation (2018M640804), the Natural Science Foundation of Guangdong Province (2017A030310671) and the Military Logistic Science Research Project (BGZ15J001).

### Competing interest statement

The authors declare no conflict of interest.

### Additional information

No additional information is available for this paper.
